# 559. Results of the implementation of a molecular pneumonia panel at a hospital in the Dominican Republic: expanding the scope of detection and management

**DOI:** 10.1093/ofid/ofad500.628

**Published:** 2023-11-27

**Authors:** Anel E Guzman-Marte, Rita A Rojas-Fermin, Ricardo Ernesto Hernandez-Landa, Ledis Reyes-Batista, Glennys S Camilo

**Affiliations:** Hospital General de la Plaza de la Salud, Santo Domingo, Distrito Nacional, Dominican Republic; Hospital General de la Plaza de la Salud, Santo Domingo, Distrito Nacional, Dominican Republic; Universidad Ibero Americana, Santo Domingo, Distrito Nacional, Dominican Republic; Universidad Nacional Pedro Henriquez Urena, santo domingo, Distrito Nacional, Dominican Republic; Universidad Nacional Pedro Henriquez -Urena, Santo Domingo, Distrito Nacional, Dominican Republic

## Abstract

**Background:**

Lower respiratory tract infections (LRTIs) have been associated to significant morbidity and mortality. Conventional microbiology methods often fail to identify the etiological agent due to lack of sensitivity or viral or fastidious pathogens. Pneumonia molecular diagnostics is able to expand the scope of pathogens detected, therefore, we describe our experience in the incorporation of this method, the difference in etiological identification and clinical decision making.

**Methods:**

We performed a retrospective cohort study of 75 patients with Filmarray pneumonia panel from November 2020 to September 2022. We described demographics, co-morbid conditions, outcomes and correlation with standard cultures in patients who were admitted with LRTI at the Hospital General de la Plaza de la Salud, a 289-bed tertiary teaching hospital in the Dominican Republic.

**Figure:**
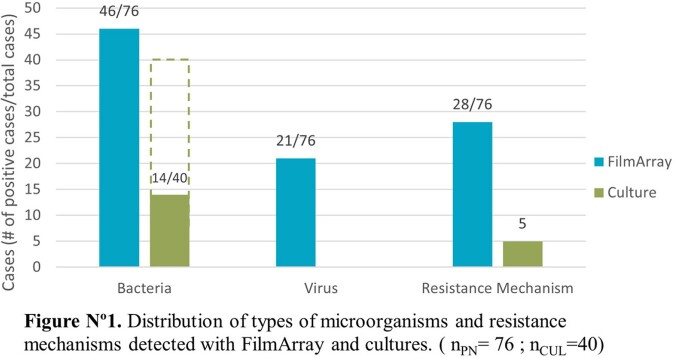

**Results:**

Amongst the 75 patients, 56.6% were male and the average age was 51 years old. The most common comorbidities were diabetes mellitus (81.6%), hypertension (40.8%) and nephropathy (10.5%). The panels had a positivity rate of 74.6% (56/75) versus a 35% (14/40) seen in cultures. The most frequent microbial targets detected were *S. aureus* (18%), *P. aeruginosa* (14,3%) and *K. pneumoniae group* (10%) and at least one antimicrobial resistance gene was detected in 50% (28/56), distributed as MecA/C and MREJ (35%), CTX-M (38%), KPC (10%), NDM (10%), VIM (8%), IMP (8%) and OXA-48 like (3%). A positive culture was most likely to occur when the number of copies/mL in the panel was ≥ 10˄5 (p-value < 0.001). A viral target was detected in 21 patients (28%) alone or coinfecting with a bacterial agent.

**Figure:**
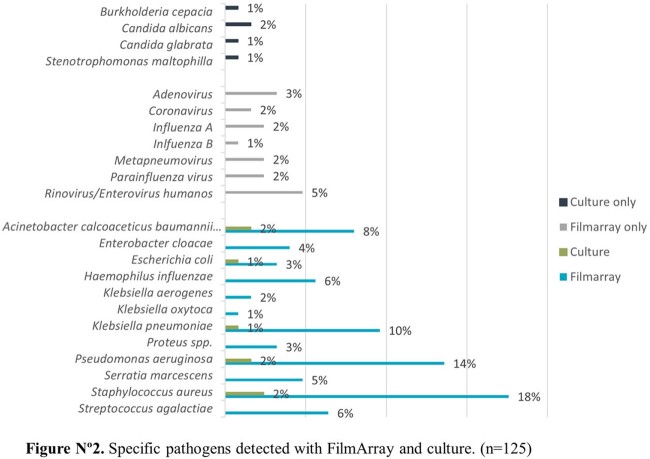

**Figure:**
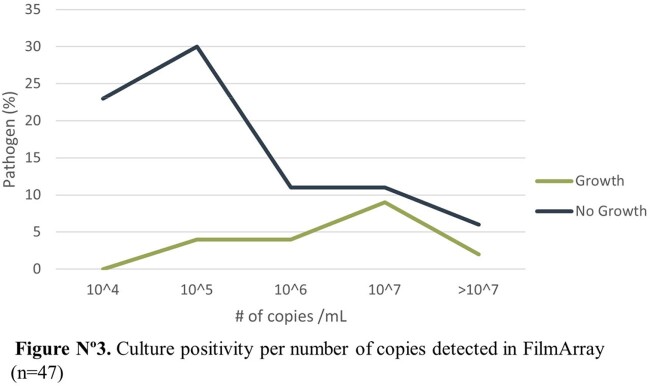

**Figure:**
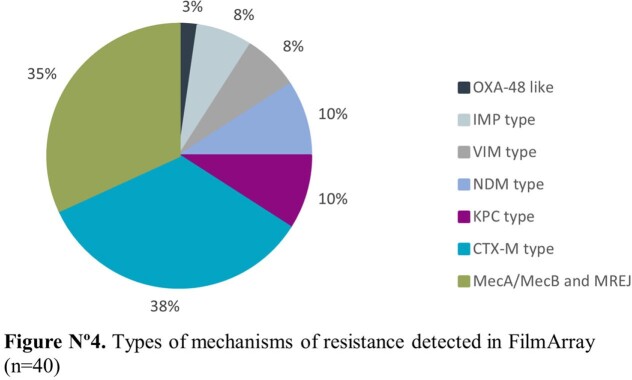

**Conclusion:**

Not only did the pneumonia panels allow an earlier detection of the etiological agent, but they also identified more pathogens compared to cultures. This enabled targeted management by reducing the unnecessary use of antimicrobials as well as activating infection control measures when necessary (i.e Influenza). The results seen in this study, along with the expanded scope of pathogens detected, will definitely serve as an update into our current LRTI hospital guidelines.

**Disclosures:**

**All Authors**: No reported disclosures

